# Optogenetics in bacteria – applications and opportunities

**DOI:** 10.1093/femsre/fuab055

**Published:** 2021-11-13

**Authors:** Florian Lindner, Andreas Diepold

**Affiliations:** Department of Ecophysiology, Max-Planck-Institute for Terrestrial Microbiology, Karl-von-Frisch-Str. 10, 35043 Marburg, Germany; Department of Ecophysiology, Max-Planck-Institute for Terrestrial Microbiology, Karl-von-Frisch-Str. 10, 35043 Marburg, Germany; SYNMIKRO, LOEWE Center for Synthetic Microbiology, Karl-von-Frisch-Straße 14, 35043 Marburg, Germany

**Keywords:** synthetic biology, optogenetic interaction switches, two-component systems, protein interactions, biotechnology, fluorescence, light-sensing domains

## Abstract

Optogenetics holds the promise of controlling biological processes with superb temporal and spatial resolution at minimal perturbation. Although many of the light-reactive proteins used in optogenetic systems are derived from prokaryotes, applications were largely limited to eukaryotes for a long time. In recent years, however, an increasing number of microbiologists use optogenetics as a powerful new tool to study and control key aspects of bacterial biology in a fast and often reversible manner. After a brief discussion of optogenetic principles, this review provides an overview of the rapidly growing number of optogenetic applications in bacteria, with a particular focus on studies venturing beyond transcriptional control. To guide future experiments, we highlight helpful tools, provide considerations for successful application of optogenetics in bacterial systems, and identify particular opportunities and challenges that arise when applying these approaches in bacteria.

## Teaser

This review highlights the diversity of optogenetic approaches in prokaryotes and aims to provides microbiologists with a guide for the application of light for precise, reversible and non-invasive control of biological processes in bacteria.

## INTRODUCTION

Optogenetics has its origin in the 1970s when researchers found that bacterial rhodopsins act as ion pumps that are activated by visible light (Oesterhelt and Stoeckenius 1971; Oesterhelt 1976). During that time, Francis Crick challenged neuroscientists to come up with a system that would allow the precise stimulation of single neurons, as opposed to the large areas stimulated by electrodes (Crick 1979; Siegel and Callaway 2004; Deisseroth 2011). At the time, this was perceived as an almost impossible task. However, a breakthrough was made in the seemingly distant field of plant and microbial biology, when the group of Peter H. Quail developed a light-controlled gene promoter (Shimizu-Sato *et al*. 2002) based on the plant phytochrome B-phytochrome interacting factor 3 (PhyB-PIF3) photoreceptor system (Ni, Tepperman and Quail 1999), and three years later, Boyden and coworkers found that expression of an algal channelrhodopsin made neurons respond to light (Boyden *et al*. 2005). Since then, optogenetics has found wide range of application in eukaryotic systems, extending from neuroscience (Boyden 2011; Yizhar *et al*. 2011) to areas as diverse as cardiology (Entcheva and Kay 2020) and regenerative medicine such as bone repair (Sato *et al*. 2018) or restoration of muscle function (Bryson *et al*. 2014).

The first optogenetic setup in bacteria was established in 2005, when Levskaya and colleagues developed a synthetic sensor kinase, based on the photoreceptor Cph1 and the histidine kinase EnvZ, to achieve red light-controllable gene expression of LacZ in *Escherichia coli* (Levskaya *et al*. 2005). Since then, the number of optogenetic applications in prokaryotes has markedly increased. This review will summarize these applications and provide an overview of the range of optogenetic approaches applied in bacteria to date. To facilitate the development of even more diverse applications of optogenetics in prokaryotes in the future, we will discuss the specific requirements, challenges, and opportunities of applying optogenetics in bacteria. A characterization of optogenetic base systems, including their light spectrum and dynamics, a list of useful tools for optogenetic setups in bacteria, and guidelines for selecting the optimal system for specific approaches are aimed at helping microbiologists evaluate these systems and design experiments to address individual biological questions. We want to draw particular attention to studies that go beyond the control of gene expression, which is currently the main application area of optogenetics in bacteria, and showcase its great potential for the direct control of diverse biological processes with light.

## OPTOGENETIC SYSTEMS

Optogenetics combines optical and genetic techniques to design and apply light-sensitive proteins in order to control cellular processes within living organisms. Light-reactive proteins or protein domains are often plant-derived, such as phytochromes, cryptochromes, light-oxygen-voltage sensing (LOV) and ultraviolet B resistance locus 8 (UVR8) domain proteins, but also originated in bacteria, archaea, algae, and higher animals, for example channelrhodopsins, halorhodopsins, cyanobacteriochromes, cryptochromes, phytochromes, and additional LOV domain proteins (Endo and Ozawa 2017). Upon illumination with a specific wavelength, the light-sensing domains undergo a conformational change. The resulting modulation of protein properties can lead to association/dissociation with an interaction partner or partial folding/unfolding of the protein structure, which then controls downstream biological processes.

Optogenetic systems constitute excellent tools for the direct control of biological systems in a highly time- and space-resolved manner with minimal intervention. They allow for easy and often reversible manipulation of protein functionality and localization (Guglielmi, Falk and De Renzis 2016), metabolism (Berry and Wojtovich 2020), intracellular interaction of enzymes and substrates (Huang *et al*. 2020), or processes like gene expression (de Mena, Rizk and Rincon-Limas 2018). Its fast and easy tunability gives light an advantage over chemical inducers and other environmental triggers like pH or temperature and enables more precise spatiotemporal control (Deisseroth 2011; Liu *et al*. 2018), even up to a single-cell level (Chait *et al*. 2017). By modulating the amplitude or pulse-width of the light source (Baumschlager and Khammash 2021) or by combining different optogenetic approaches (Tabor, Levskaya and Voigt 2011; Fernandez-Rodriguez *et al*. 2017), even more complex modulation of expression can be achieved. Different optogenetic systems react to light across the whole visible spectrum and extending into the infrared range, which can be preferable in sensitive systems due to its lower energy. While the vast majority of applications were originally developed in eukaryotes, optogenetics increasingly finds its way into prokaryotic systems.

**Figure 1.**
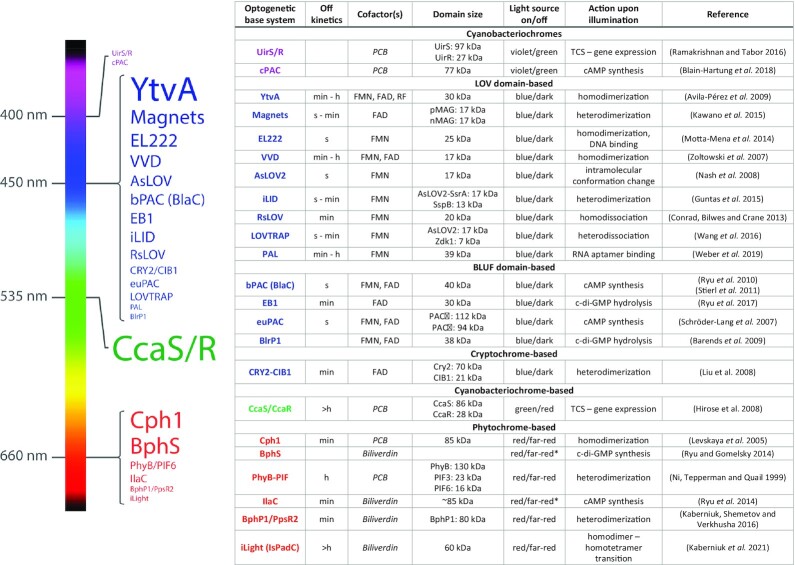
Optogenetic base systems used in bacteria Left, graphical representation of main optogenetic base systems used in bacteria. Activation wavelength and position in visible spectrum indicated; word size represents number of applications (font size ∼ square root of number of applications listed for bacterial host in optobase.org (Kolar et al. 2018) for all systems with applications in bacteria as of August 2021). Right, key properties of these optogenetic base systems. FMN, Flavin mononucleotide; FAD, flavin adenine dinucleotide; RF, riboflavin; PCB, phycocyanobilin. *, far-red-induced inactivation was shown for BphG, which BphS and IlaC are derived from (Tarutina, Ryjenkov and Gomelsky 2006), not for the systems themselves. Italics: cofactor not ubiquitously present in bacteria; may need to be synthesized.

In this review, we define *optogenetic base systems* as domains, proteins or pairs thereof that react to light and are modified and applied for optogenetic applications. In most cases, the direct reaction to illumination occurs in a bound chromophore cofactor, which features the conjugated electron system that can absorb photons. The resulting change in conformation is then transmitted to the protein core. Optogenetic base systems comprise a diverse collection of proteins, which can be generally divided into channel proteins, widely used in neurobiology, and intracellular proteins, which are the predominant class for applications in prokaryotes. Channel systems are mostly based on opsins, which are light-driven ion pumps or channels carrying the chromophore retinal, or on opsin variants (channelrhodopsins and halorhodopsins) (Terakita 2005; Fenno, Yizhar and Deisseroth 2011). Intracellular optogenetic base systems include proteins from the LOV, blue-light-utilizing flavin adenine dinucleotide (BLUF), cryptochrome (CRYs) (all reacting to blue light), phytochrome (PHY, red-light-responsive), and UVR8 (reacting to ultraviolet light) families (Van Der Horst and Hellingwerf 2004; Möglich and Moffat 2010; Pudasaini, El-Arab and Zoltowski 2015). These base systems can then be either adapted or simply combined with other proteins or domains in a modular manner, extending light control to usually non-light-reactive proteins. An excellent example for this approach is the development of the light-responsive histidine kinase YF1, part of a much-used optogenetic two-component system (TCS) for transcriptional regulation. Möglich and colleagues structurally aligned a LOV-based photoreceptor, *Bacillus subtilis* YtvA (Losi *et al*. 2002) and an oxygen-sensing histidine kinase, *Bradyrhizobium japonicum* FixL, both of which feature N-terminal sensor and C-terminal output domains. Testing different recombination points in a central α-helical domain, they identified fusion proteins conferring light-dependent activation of the cognate response regulator FixJ (Möglich, Ayers and Moffat 2009). The resulting TCS YF1/FixJ has since been evolved further to allow for bidirectional control of protein expression (see chapter 3.1.2). Fig. 1 provides an overview of optogenetic base systems that have been used in bacteria and their respective activation wavelength, which covers the visible spectrum from violet to red with a particular density of systems reacting to blue light. Readers are encouraged to refer to specialized reviews (Shcherbakova *et al*. 2015; O'Banion and Lawrence 2018) for details on the biophysical mechanisms of these and other base systems.

## OPTOGENETIC APPLICATIONS IN BACTERIA

The modularity of optogenetic systems, which allows control of protein behavior by combining proteins of interest (POI) with light-reactive base systems, has led to the development of a large variety of different systems, many of which build on each other through both linear evolution and combination.

**Figure 2.**
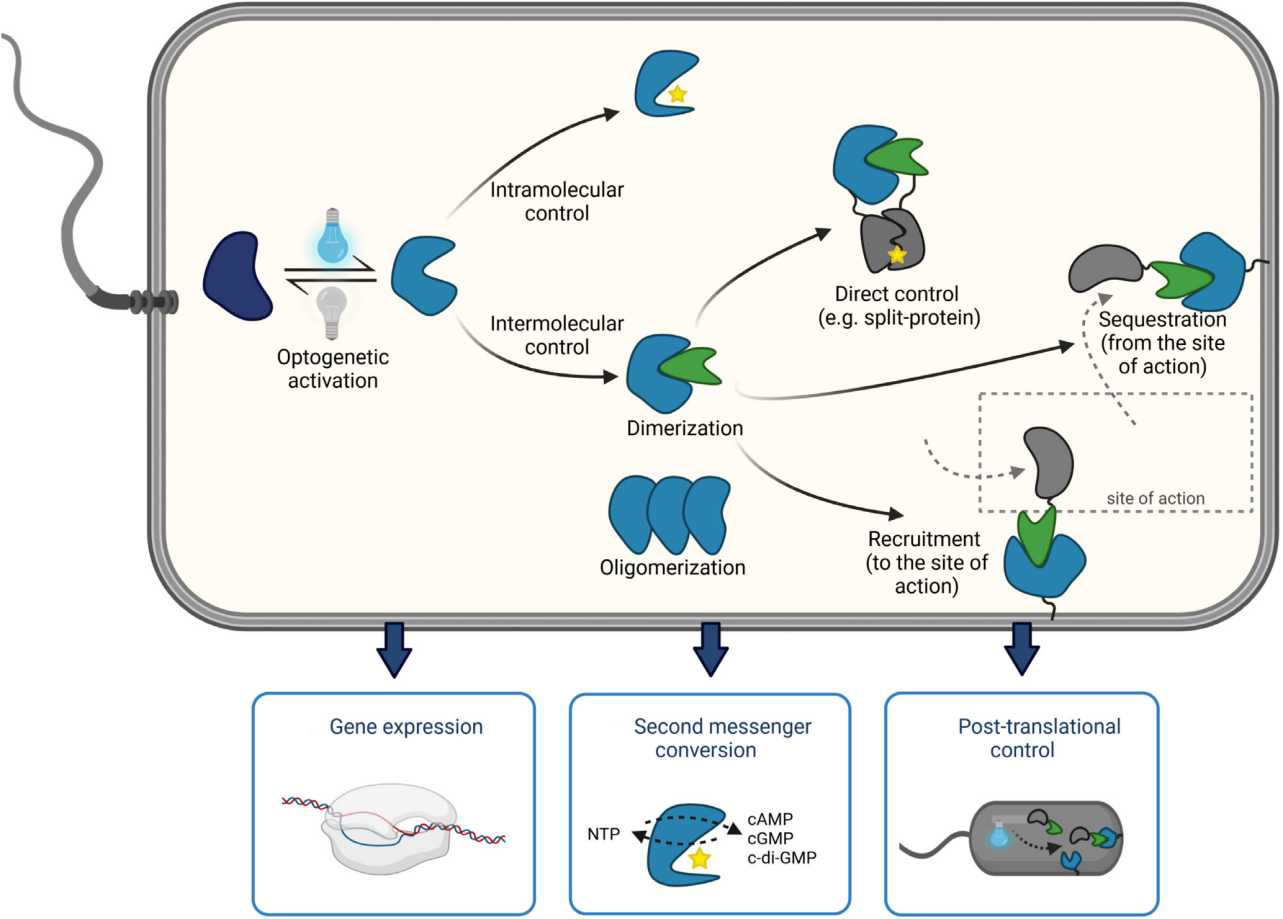
Optogenetics in bacteria Main pathways of optogenetic control in bacteria (top), and resulting application classes (boxes below). Note that application classes are grouped according to the outcome rather than the mechanism of action of the optogenetic system. Figure created with biorender.com; see main text for details.

Optogenetic applications can be roughly grouped into intra- and intermolecular systems. While intramolecular systems consist of only one light-sensitive protein, intermolecular systems have additional interaction partners (Fig. 2).

In intramolecular systems, absorption of light leads to a conformational change in the light-sensitive part of the protein, which is transmitted intramolecularly to an effector domain and modulates the conformation or accessibility of an active center, a substrate-binding pocket, or a regulatory domain. The design of intramolecular optogenetic systems requires knowledge about the behavior of the optogenetic base system as well as the POI's structure-function relationship (see paragraph “Intramolecular control" for details).

Intermolecular systems, also called optogenetic interaction switches, consist of two or more molecules whose binding is influenced by a conformational change in at least one of the interaction partners, in analogy to chemical dimerizers (Fegan *et al*. 2010). These systems mostly exploit light-induced homo- and hetero-dimerization or -dissociation, although examples of light-induced oligomerization exist. In this review, ‘optogenetic interaction switch’ will be used exclusively for the control of intermolecular interactions, which can be used to directly activate (or, in special cases, inactivate) protein function via dimerization or oligomerization, to recruit a POI to its site of action, or, conversely, to sequester it from its site of action, such as the cytosol (Fig. 2).

Related aims can be achieved with different optogenetic systems. A prime example is the large field of transcriptional control (discussed in the next paragraph), which has been accomplished by intra- and intermolecular optogenetic systems. Similarly, direct protein control (see paragraph “Direct (post-translational) control of protein function by illumination") can be achieved by intramolecular optogenetic actuators, although most published systems rely on intermolecular interactions, such as optogenetic interaction switches.

In the last years, the utilization of optogenetics to investigate biological processes in bacteria has markedly increased (Table 1). To date, the most established application of optogenetic systems in prokaryotes is the light-mediated control of gene expression.

**Table 1.** Optogenetic applications in bacteria. Optogenetic applications in prokaryotes. Examples are subsequently ordered by the type of control mechanisms (subheadings), light range, optogenetic system and chronologically. Where applicable, the left column indicates both the modification of the system and the application, separated by a dash. The central column indicates the optogenetic system used and, where applicable, the base system, according to the classification used in Fig. 1, which used the optobase.org classification (in brackets) and the controlled protein or domain (orange font).

Application in bacteria	Bacterial host	Optogenetic system (base system)*Controlled domain/protein*	Light source on/off	Reference
***Control of gene expression***				
Development of violet/green gene control circuit	*E.coli*	UirS/R	violet/green	(Ramakrishnan and Tabor 2016)
Development of optogenetic sensor kinase YF1 for gene expression	*E. coli*	YF1/FixJ (YtvA)	blue/dark	(Möglich et al. 2009)
Development of optogenetic pDusk/pDawn gene expression plasmid for bidirectional control of expression	*E. coli*	pDawn; pDusk (YtvA)	blue/dark; dark/blue	(Ohlendorf et al. 2012)
Expression of ß-glucosidase coupled with induced cell lysis—biochemical production	*E. coli*	pDawn (YtvA)	blue/dark	(Chang et al. 2017)
Expression of murein hydrolase—controlled cell lysis	*E. coli*	pDusk (YtvA)	blue/dark	(Wang et al. 2018)
Expression of adhesin Ag43–control of biofilm formation	*E. coli*	pDawn (YtvA)	blue/dark	(Jin and Riedel-Kruse 2018)
Expression of phosphodiesterase—control of biofilm degradation	*P. aeruginosa*	pDawn (YtvA)	blue/dark	(L. Pu et al. 2018)
Expression of antitumoral and antimicrobial drug deoxyviolacein	*E. coli*	pDawn (YtvA)	blue/dark	(Sankaran et al. 2019)
Part of synthetic biology toolbox for *V*. *natriegens*	*V. natriegens*	pDawn (YtvA)	blue/dark	(Tschirhart et al. 2019)
Secretion of probiotics in upconversion system allowing near-infrared (NIR) activation	*E. coli* *L. lactis*	pDawn (YtvA)	blue/dark (NIR by upconversion)	(Yang et al. 2020)
Optogenetic regulation of the lac operon (OptoLAC)—biochemical production	*E. coli*	pDawn (YtvA)	blue/dark	(Lalwani et al. 2021)
Development of optogenetic gene expression circuit (EL222)—control by pulsed illumination	*E. coli*	EL222	blue/dark	(Jayaraman et al. 2016)
Expression of CheZ—control of bacterial directional motility	*E. coli*	EL222	blue/dark	(Zhang et al. 2020)
Expression switch between ribonucleotide reductase NrdAB and division proteins FtsZ/A (BARNA)—control of cell cycle duration	*E. coli*	EL222; BphS	blue/red/dark	(Ding et al. 2020)
Expression of EPS—control of biofilm structures	*S. meliloti*	EL222	blue/dark	(Pirhanov et al. 2021)
Expression of dCpf1-mediated CRISPRi—switch between growth and muconic acid synthesis	*E. coli*	EL222	blue/dark	(Wu et al. 2021)
Translational control using light-dependent RNA binding of *Nakamurella multipartite* photoreceptor PAL	*E. coli*	PAL	blue/dark	(Weber et al. 2019)
Expression of FtsZ and CheZ (eLightOn)—control of cell division or motility	*E. coli*	RsLOV LexRO	blue/dark	(Li et al. 2020)
Control of virulence effector expression—infection of *C. elegans*	*P. aeruginosa*	YtvA GacS	blue/dark	(Cheng et al., 2021)
Development of light-controlled split T7-RNAP	*E. coli*	iLID T7-RNAP	blue/dark	(J. Pu et al. 2017)
Development of light-controlled split T7-RNAP	*E. coli*	Magnets T7-RNAP	blue/dark	(Baumschlager et al. 2017)
(Anhydro)tetracycline (aTC/TC) as photoactivatable compounds for expression and growth control—enhanced expression control (split-T7-RNAP)	*E. coli*	aTC/TC; Magnets T7-RNAP	blue/dark	(Baumschlager et al. 2020)
Development of split T7-RNAP—control gene expression either by dimerization or allosteric blocking	*E. coli*	VVD; Magnets T7-RNAP	blue/dark	(Han et al. 2017)
Development of optogenetic DNA repressor LexA (LEVI)—high on/off ratio—control of induced cell death (CcdB), motility (CheZ) and biochemical production	*E. coli*	VVD LexA	blue/dark	(X. Chen et al. 2016)
Development of optogenetic AraC-based expression system (BLADE)	*E. coli*	VVD AraC	blue/dark	(Romano et al. 2021)
Multichromatic control of gene circuit—red (Cph8), green (CcsAR), blue (YtvA)—control of gene expression and metabolic flux	*E. coli*	YtvA; CcaS/R; Cph8	blue/green/red/dark	(Fernandez-Rodriguez et al. 2017)
Development of a two-color optical gene expression control system	*E. coli*	CcaS/R; Cph8	green/red; red/far-red	(Tabor et al. 2011)
Gene expression system in cyanobacteria	*Synechocystis sp*.	CcaS/R	green/red	(Abe et al. 2014)
Expression of T4 phage holin, endolysin—controlled bacterial lysis and release of phycocyanin into medium for chemical production	*Synechocystis sp*.	CcaS/R	green/red	(Miyake et al. 2014)
Enhanced TCS expression system in *E. coli*—modified synthetic promotors to increase dynamic range	*E. coli*	CcaS/R; Cph8 (Cph1)	green/red; red/far-red	(Schmidl et al. 2014)
Expression of adhesin Ag43–light-mediated cell recovery system (self-aggregation)	*E. coli*	CcaS/R	green/red	(Nakajima et al. 2016)
Modified CcaS/R system—(PAS domain double deletion)—four times lower off-leakiness and 600‐fold dynamic expression range	*E. coli*	CcaS/R	green/red	(Ong and Tabor 2018)
Flux control between glycolysis and the methylglyoxal pathway	*E. coli*	CcaS/R	green/red	(Senoo et al. 2019)
Adaptation of CcaS/R gene circuit to *B. subtilis—*enhanced and modified PCB synthesis via translational fusion	*B. subtilis*	CcaS/R	green/red	(Castillo-Hair et al. 2019)
Regulation of glycolytic flux	*E. coli*	CcaS/R	green/red	(Tandar et al. 2019)
Adaptation of CcaS/R gene circuit to *P. putida—*PCB synthesis, CcaS/R and cognate downstream promotor on single vector	*P. putida*	CcaS/R	green/red	(Hueso-Gil et al. 2020)
Expression of colonic acid—secreted by *E. coli* in gut, affects *C. elegans* longevity	*E. coli*	CcaS/R	green/red	(Hartsough et al. 2020)
Development of a red-light gene control system based on Cph1 and histidine kinase EnvZ	*E. coli*	Cph8 (Cph1)	red/far-red	(Levskaya et al. 2005)
Combination of direct and diffusion-based optogenetic control—dark/light edge detection by expression of black pigment	*E. coli*	Cph8 (Cph1)	red/far-red	(Tabor et al. 2009)
Engineered Cph1-based histidine kinase library—control of gene expression	*E. coli*	Cph1	red/dark	(Ma et al. 2017)
Development of different TCS (controllable with light, nitrate, glucose)	*E. coli*	Cph8 (Cph1)	red/far-red	(Schmidl et al. 2019)
Development of NIR-controlled OCS system (iLight)—smaller and packable in adenovirus	*E. coli*	iLight	red/NIR	(Kaberniuk et al. 2021)
Development of a light-activatable split-intein T7-RNAP—control of lycopene synthesis	*E. coli*	PhyB/PIF3 Split-VMA intein	red	(Raghavan et al. 2020)
Development of most red light shifted gene control system	*E. coli*	BphP1/PpsR2	NIR/red	(Ong et al. 2018)
				
***Second messenger conversion***				
First description of blue-light activatable adenylate cyclase (BlaC) from Beggiatoa—adaptation of guanylate cyclase (BlgC) for cAMP/cGMP synthesis	*E. coli*	BlaC (bPAC)	blue/dark	(Ryu et al. 2010)
Blue-light activatable adenylate cyclase (bPAC) from the soil bacterium Beggiatoa—control of cAMP synthesis	*E. coli*	bPAC	blue/dark	(Stierl et al. 2011)
cAMP synthesis module for *Pseudomonas*—control of twitching motility and host cell infection (mouse)	*P. aeruginosa*	bPAC	blue/dark	(Xia et al. 2021)
Development of cyanobacteriochrome-based photoswitchable adenylyl cyclase (cPAC)—control of cAMP synthesis—LacZ expression as readout	*E. coli*	cPAC	blue/green	(Blain-Hartung et al. 2018)
Conversion of c-di-GMP—characterization of aerotaxis in Azospirillum *brasilense*	*A. brasilense*	BphS; EB1	NIR/blue	(O'Neal et al. 2017)
Conversion of c-di-GMP—regulation of motility and biofilm in *E. coli* and chemotaxis in A. brasilense	*E. coli; A. brasilense*	BphS; EB1	NIR/blue	(Ryu et al. 2017)
Conversion of c-di-GMP—bioprinting defined structures of bacterial biofilm	*P. aeruginosa*	BphS; BlrP1	NIR/blue	(Y. Huang et al. 2018)
Conversion of c-di-GMP—control of biofilm for mitigating biofouling and water purification purpose	*E. coli*	BphS; EB1	NIR/blue	(Mukherjee et al. 2018)
Synthesis of c-di-GMP—control of biofilm formation to catalyze the biotransformation of indole into tryptophan	*E. coli*	BphS	NIR/dark	(Hu et al. 2019)
				
***Post-translational control***				
Proof of concept for engineering light-regulatory activities by interface design at conserved allosteric sites—light-activatable dihydrofolate reductase	*E. coli*	*As*LOV2 DHFR	blue/dark	(Lee et al. 2008)
Development of optogenetic Cas9–allosteric inhibition by dimerization (dark), release and activation (light)—induced DNA cleavage activity	*E. coli*	*Rs*LOV Cas9	blue/dark	(Richter et al. 2013)
Oligomerization of intein domains mediated through conformational change—functional protein self-splicing and activation	*E. coli*	*As*LOV2 *Npu* intein	blue/dark	(Jones et al. 2016)
Formation of cytotoxic oligomers—control amyloidogenesis—control arrest of cell growth	*E. coli*	*As*LOV2 WH1	blue/dark	(Giraldo 2019)
Light-induced non-apoptotic tools (LIPOPS) based on dimerization—control of cell death	*E. coli*	*As*LOV2; CRY2	blue/dark	(He et al. 2021)
Light-controlled enrichment of proteins in cytosolic compartments formed by liquid-liquid phase separation—up to 15-fold enrichment of POI	*E. coli*	CRY2/CIB1s	blue/dark	(Z. Huang et al. 2020)
Optogenetic protein expressed on outer surface of bacteria—attachment to optogenetic interaction protein containing surface—control of biofilm formation	*E. coli*	Magnets	blue/dark	(F. Chen and Wegner 2017) (F. Chen and Wegner 2020)
Optogenetic protein expressed on outer surface of bacteria—control of aggregation, Hg^2+^ sensing and biofilm formation	*E. coli*	Magnets	blue/dark	(F. Chen et al. 2020)
Split-recombinase for DNA modification	*E. coli*	VVD; Magnets Cre; Flp	blue/dark	(Sheets et al. 2020)
Optogenetic mediated oligomerization of multimeric structures—assembly of dodecamer nitrilase	*E. coli*	iLID	blue/dark	(Yu et al., 2017)
Membrane tethering of essential component of the type III secretion system—spatiotemporally control of protein delivery into eukaryotic host cells	*Y. enterocolitica*	LOVTRAP; iLID SctQ	blue/dark	(Lindner et al. 2020)
Membrane tethering of POI for protein purification	*E. coli*	iLID	blue/dark	(Tang et al. 2021)
Optogenetic protein expressed on outer surface of bacteria—attachment, transport and release of cargo proteins	*E. coli*	PhyB/PIF6	red/far-red	(Sentürk et al. 2020)

### Control of expression

Optogenetic systems regulating the expression level of proteins can be divided into one-component systems (OCS) and two-component systems (TCS). OCS comprise different optogenetic base systems designed to mediate expression control, for example by light-dependent dimerization and recruitment of transcription repressors or activators, or direct mediation of translational control (on the RNA level) by steric blocking. TCS consist of a light-activatable histidine kinase (HK) and a corresponding response regulator (RR) that is phosphorylated by the HK upon illumination and regulates downstream processes like gene expression (Schmidl *et al*. 2014). The vast majority of published optogenetic approaches for expression control target gene transcription; readers that are specifically interested in this aspect are encouraged to also refer to the excellent recent review from Baumschlager and Khammash (Baumschlager and Khammash 2021).

#### One-component systems

Blue-light mediated control over protein binding or dimerization most often relies on LOV domains due to their relatively small size (17–20 kDa), robustness and well-studied properties (reviewed in Zayner and Sosnick 2014; Pudasaini, El-Arab and Zoltowski 2015). EL222, one of the most frequently applied one-component blue light-responsive bacterial transcription factors, combines an N-terminal LOV domain with a C-terminal helix-turn-helix (HTH) DNA-binding domain (Motta-Mena *et al*. 2014; Jayaraman *et al*. 2016), which allows for the direct control of gene expression by binding to DNA upon illumination. EL222-mediated gene expression was established in a wide range of applications. Controlled expression of CheZ, a regulator of flagellar rotation, resulted in negative phototaxis in *E. coli* (Zhang, Luo and Poh 2020), while bacterial division and cell shape were perturbed by control of expression of FtsZ, a key player in bacterial cell division (Ding *et al*. 2020). In *Sinorhizobium meliloti*, EL222-controlled exopolysaccharide (EPS) synthesis was used to control the resulting biofilm formation (Pirhanov *et al*. 2021).

By fusing a LOV domain from *Rhodobacter sphaeroides* (RsLOV) to a transcriptional repressor, a different blue light-controllable gene expression system (eLightOn) was designed and, similar to the cases above, used to influence bacterial motility and cell division, respectively (Li *et al*. 2020). Romano and colleagues combined the LOV-derived VVD domain with the transcriptional regulator AraC in order to make it responsive to blue light, and created a set of blue-light-inducible AraC dimerization-based systems for expression control in *E. coli* (BLADE) (Romano *et al*. 2021). As a proof of concept, this system was used to light-control the arabinose pathway itself (Romano *et al*. 2021). By fusing the VVD domain to the *E. coli* DNA repressor LexA, an optogenetic setup for blue-light mediated gene control (LEVI) with a high on/off ratio (up to 10 000-fold) was developed. As applications, light-induced cell death (expression of the toxic protein CcdB) and motility control (expression of CheZ) were presented (Chen *et al*. 2016).

Based on a phytochrome from *Idiomarina*, a small near infrared (NIR) one-component system termed iLIGHT was developed that can be packed in adeno-associated viruses for easy transduction of eukaryotic cells and enabled light controlled gene expression with up to 115-fold dynamic range in bacteria and mouse tissue (Kaberniuk *et al*. 2021).

In a light-controlled, though not fully optogenetic approach^1^, Chou and colleagues used a photoactivatable chemical *ortho*-nitrobenzyl caging group to block the active side of T7 RNA polymerase (RNAP) in *E. coli*. Upon UV-light illumination, the caging group was released and T7 polymerase activity restored (Chou, Young and Deiters 2010). A disadvantage of this setup is the irreversibility of the reaction, unlike for photoactivatable protein domains. Therefore, Pu and colleagues engineered a split-T7 RNA polymerase (split-T7-RNAP) where two non-functional polymerase domains were fused to different optogenetic interaction switches including the pair of *Avena sativa* LOV2 (iLID) and SspB_Nano (iLID system, (Guntas *et al*. 2015)). Illumination with blue light induced dimerization of the interacting domains, restoring T7-RNAP function and activating gene expression (Pu, Zinkus-Boltz and Dickinson 2017). Han and colleagues developed a similar split-T7-RNAP setup to control gene expression by either dimerization (Magnets) or allosteric blocking (VVD) (Han, Chen and Liu 2017). To further improve previous setups, a Magnet-based split-T7-RNAP setup was combined with photodegradable compounds (anyhydrotetracycline/tetracycline) to increase optogenetic controllability and provide an optochemical gene expression system in *E. coli* (Baumschlager, Rullan and Khammash 2020). Based on a fusion of the red-light controllable PhyB-PIF3 dimerization switch with a split-intein and a split-T7-RNAP, Raghavan and colleagues accomplished a red light (∼660 nm)-induced dimerization that activated the intein self-splicing effect and released the restored and functional T7-RNAP. This application was established for the biochemical production of lycopene in *E. coli* (Raghavan, Salim and Yadav 2020).

Finally, optogenetic control of gene expression can also be achieved by influencing protein binding to RNA—the *Nakamurella multipartita* photoreceptor PAL combines a LOV sensor domain and an RNA-binding domain to bind short RNA stem-loops upon illumination, which decreases expression of the corresponding reporter gene up to 85% (Weber *et al*. 2019).

#### Two-component systems

Two-component systems usually consist of a membrane-bound histidine kinase (HK) sensing an environmental stimulus and a corresponding response regulator (RR) mediating a cellular response. The first two-component optogenetic gene expression module was designed by combining the photoreceptor Cph1 from *Synechocystis* sp. with the *E. coli* HK EnvZ. The resulting light-controllable HK Cph8 allowed for red light-controllable gene expression of *LacZ*, controlled by the cognate RR OmpR in *E. coli* (Levskaya *et al*. 2005). This TCS was also used for controlled LuxR expression within a bacterial edge detection setup, where *E. coli* were engineered to detect the border between dark and light conditions in a bacterial lawn, in analogy to edge detection algorithms in image processing (Tabor *et al*. 2009). In this landmark approach, expression of a black pigment was induced by the combination of two factors: direct illumination (more precisely the absence of darkness, implemented by the dark-activated transcription of the λ-phage repressor cI, which itself negatively controls expression of the transcription factor LuxR) and proximity to darkness (indicated by presence of the diffusible quorum signal and LuxR activator 3-oxohexanoyl-homoserine lactone (AHL), produced by siblings in the dark) (Tabor *et al*. 2009). Researchers highlighted the range of RR DNA-binding domains within TCS by domain-swapping to create, amongst others, a modified system with 1300-fold dynamic range (Schmidl *et al*. 2019). Ma and colleagues used the photoreceptor Cph1 to develop an engineered HK library, consisting of 16 HK-RR TCS to serve as a toolbox for red-light controllable gene expression in *E. coli* and other bacteria (Ma *et al*. 2017).

Based on the YtvA-based TCS YF1/FixJ , a one-plasmid system was developed that allows blue-light mediated transcriptional control in both directions, repression (pDusk) and activation (pDawn). In the latter, insertion of the λ-phage repressor cI into the pathway inverts signal polarity and allows light-activatable gene expression (Ohlendorf *et al*. 2012). Wang and colleagues used the pDusk plasmid to achieve blue-light mediated expression of lysin, a bacteriophage-derived protein disrupting the bacterial cell wall, for controlled cell lysis in *E. coli* (Wang *et al*. 2018). Other researchers modulated biofilm formation by light-dependent expression of either the biofilm degrading phosphodiesterase PA2133 in *P. aeruginosa* (Pu *et al*. 2018) or the outer membrane adhesin Ag43 in *E. coli* (Jin and Riedel-Kruse 2018). pDawn was further established as part of a synthetic toolbox for bioengineering purposes in *Vibrio natriegens* (Tschirhart *et al*. 2019) and for applications with a focus on biochemical production (Box 1). Optogenetic regulation of gene expression with TCS has also been applied in infection biology, where a blue light-controllable sensor kinase, a chimera of a LOV domain and the native sensor kinase GacS, was used to control the expression of virulence factors in *P. aeruginosa* and subsequently modulate infection in the model system *Caenorhabditis elegans* (Cheng *et al*. 2020).

**Box 1. Applying optogenetics for biological compound production.**

Optogenetic approaches are increasingly used to control microbial industrial production, where a tight balance between culture growth phase and bioproduction phase is necessary to maximize production (reviewed in Pouzet *et al*. 2020). Optogenetic control of bioproduction is often based on light-controlled gene expression with the presented gene circuits pDawn (Ohlendorf *et al*. 2012), EL222 (Motta-Mena *et al*. 2014) or other TCS that enable a switch between bioproduction and growth phase by changing the light conditions (Senoo *et al*. 2019; Wu *et al*. 2021). Light-mediated applications in microbial bioproduction include metabolites like muconic acid (Wu *et al*. 2021), mevalonate or isobutanol (Lalwani *et al*. 2021); important enzymes, such as ß-glucosidase (Chang *et al*. 2017); or antimicrobial drugs (Sankaran *et al*. 2019). Some applications allow control of the metabolic flux by switching between two different glycolytic pathways (Tandar *et al*. 2019) or between glycolysis and the methyglyoxal pathway (Senoo *et al*. 2019). To automate and optimize optogenetic-driven bioproduction in bacteria, bioreactor devices that allow cultivation, illumination and real-time feedback control for the equilibrium between growth and production phase were developed (Milias-Argeitis *et al*. 2016; Wang and Yang 2017). The optogenetic control of bioproduction is sometimes also coupled to induced microbial lysis, to provide an efficient release of the product into the medium (Miyake *et al*. 2014; Chang *et al*. 2017).

One of the most widely used systems is CcaS/R, a modified cyanobacterial TCS comprising a light-regulated HK that is activated and deactivated in response to green and red light, respectively, to phosphorylate its cognate RR and transcription activator CcaR (Tabor, Levskaya and Voigt 2011). This TCS was also applied in other bacteria like the cyanobacterium *Synechocystis sp*. (Abe *et al*. 2014), *Pseudomonas putida* (Hueso-Gil *et al*. 2020), and *Bacillus subtilis* (Castillo-Hair *et al*. 2019), and further modified to achieve an increased dynamic range (up to 600-fold for gene expression control in *E. coli*) by reducing leakiness under ‘off’ conditions (Ong and Tabor 2018). CcaS/R-mediated gene expression control was used for diverse applications in bacteria like the regulation of metabolic flux (Senoo *et al*. 2019; Tandar *et al*. 2019) (see Box 1 for further details), light-controlled bacterial lysis (Miyake *et al*. 2014), easier harvesting of bacteria by expression of a self-aggregating antigen (Nakajima *et al*. 2016), and for colanic acid production in *E. coli*, where the optogenetic control allowed for the controlled synthesis in the *C. elegans* gut, resulting in a beneficial effect on gut metabolism and longer worm life (Hartsough *et al*. 2020).

TCS that can be controlled with ultraviolet (UV) light, such as UirS/R (Ramakrishnan and Tabor 2016) were designed for a wide spectral range of orthogonal light-controllable TCS and for multichromatic control of gene expression (Tabor, Levskaya and Voigt 2011; Schmidl *et al*. 2014; Fernandez-Rodriguez *et al*. 2017). To achieve an even more red light-shifted two-component-like optogenetic gene control system, the *Rhodopseudomonas* bacteriophytochrome BphP1 and its cognate RR PpsR2 were modified; near infra-red light at ∼760 nm activates BphP1, which then binds the transcription repressor PpsR2 and activates gene expression in *E. coli*, while red light at ∼660 nm accelerates the return to the ‘off’ state (Ong, Olson and Tabor 2018).

### Control of second messenger conversion

About ten years ago, a light-dependent adenylate cyclase (bacterial photoactivated adenylyl cyclase, bPAC) containing a BLUF domain was identified and isolated from the soil bacterium *Beggiatoa* and integrated into *E. coli* for blue light-dependent cyclic adenosine monophosphate (cAMP) synthesis, a key tool for future investigations of signal transduction pathways (Ryu *et al*. 2010; Stierl *et al*. 2011). By mutating critical residues of this enzyme, Ryu and colleagues further developed a blue light-controllable guanylate cyclase (BlgC) for light-mediated synthesis of cyclic GMP (cGMP) in *E. coli* (Ryu *et al*. 2010). Light-dependent cAMP synthesis, mediated by bPAC, was later applied in *P. aeruginosa* to control twitching motility and host cell infection (Xia *et al*. 2021). The development of a cyanobacteriochrome-based photoswitchable adenylate cyclase (cPAC) that allows activation and deactivation with blue and green light, respectively, further extended the optogenetic toolbox of controlled cAMP synthesis in bacteria (Blain-Hartung *et al*. 2018). To generate a regulatory c-di-GMP synthesis and degradation module, a NIR light-responsible diguanylate cyclase (BphS) (Ryu *et al*. 2014) and a counteracting phosphodiesterase EB1 (Ryu *et al*. 2017), responding to blue light, were combined with the aim of controlling biofilm formation in *E. coli* in a bidirectional manner (Ryu *et al*. 2017; Mukherjee *et al*. 2018) and chemo-/aerotaxis in *Azospirillum brasilense* (O'Neal *et al*. 2017; Ryu *et al*. 2017). The resulting control over biofilm production was then in turn used to catalyze the biotransformation of indole into tryptophan (Hu *et al*. 2019). Huang and colleagues designed a similar optogenetic setup, using another phosphodiesterase (BlrP1) for blue light-controlled c-di-GMP synthesis in *P. aeruginosa* to induce bioprinting of defined structures of bacterial biofilms with high spatial resolution (∼10 µm) (Huang *et al*. 2018).

### Direct (post-translational) control of protein function by illumination

Beyond the control of gene expression or second messenger conversion, researchers become increasingly interested in using light to control the localization and function of matured target proteins. A key advantage of this approach is that by bypassing the time needed for gene transcription and protein expression, it offers faster and more direct control over biological processes, compared to optogenetically controlled gene expression (Liu *et al*. 2018). There are a few striking examples of POIs that were engineered for intramolecular light response; however, most applications of direct optogenetic control are based on homo- or heterodimerization events.

#### Intramolecular control

As mentioned earlier, the design of intramolecular control systems requires knowledge about both the optogenetic base and the output system. LOV2 variants are frequently used as light-sensitive parts due to their strong and well-defined conformational change upon illumination. Activation of the light-sensitive flavin mononucleotide (FMN) cofactor associated to LOV2 leads to the formation of a metastable covalent link to a cysteine residue in the LOV2 domain. This event triggers the release of the C-terminal Jα helix of LOV2 (Harper, Neil and Gardner 2003; Harper, Christie and Gardner 2004; Halavaty and Moffat 2007; Peter, Dick and Baeurle 2010). The structural rearrangement of this terminal region, which can be easily attached to an output protein domain, can induce unfolding, dimerization or tilting of the output protein. Together with the considerable shift in equilibrium free energy between the two states (3–4 kcal/mol), this facilitates the application of LOV2 for intramolecular light response systems (Herrou and Crosson 2011; Zayner, Antoniou and Sosnick 2012).

In a groundbreaking proof of concept for engineering light-regulatory activities into proteins through interface design at allosteric sites, a light-activatable dihydrofolate reductase (DHFR) was designed in *E. coli*, by coupling it to the photoactivatable LOV2 domain of *Avena sativa*. Upon blue light illumination, the LOV domain, located between two DHFR domains, undergoes a conformational change that leads to a small, but significant (1.6–2.0-fold) change in DHFR catalysis rate (Lee *et al*. 2008). In a variation of this approach, Jones and colleagues fused a LOV domain to a split intein-extein module to achieve controlled self-splicing upon blue-light-triggered conformational change of the LOV2 linker protein, followed by the release of the functional extein (POI) (Jones, Mistry and Tavassoli 2016). The combination of split-inteins with LOV also allows trans-splicing, which can be used for the light-regulated recombination of mature fusion proteins (such as swapping a membrane-bound and a cytosolic protein domain) (Wong, Mosabbir and Truong 2015).

#### Intermolecular control

Light-induced conformational change can be used to regulate the affinity to other proteins, which allows the optogenetic control of protein di- and oligomerization. To date, there are few examples where the interaction of more than two proteins was exploited: LOV2-dependent oligomerization of the prion-like WH1 protein upon blue light illumination in *E. coli* was used to induce the irreversible formation of cytotoxic amyloid oligomers, which lead to growth arrest (Giraldo 2019), while the oligomerization of CRY2-linked receptor-interacting protein kinases upon illumination (termed light-induced non-apoptotic tools, LIPOPS) initiated pathways leading to killing of bacteria, as well as necroptosis and pyroptosis, which was used to reverse tumor progression in a mouse model (He *et al*. 2021).

Most post-translational optogenetic applications in bacteria are based on dimerization events, where one interacting protein (often referred to as bait) is reversibly recruited to its partner (anchor) upon illumination. Dimerization can then directly influence protein activity, or can be used to relocalize a protein to its fixed interaction partner, which in turn can activate (recruitment) or inactivate (sequestration) the POI (Fig. 2). As mentioned earlier, engineering a protein to directly influence its activity by illumination requires knowledge about its structure-function relation, which so far has limited the number of applications. Using the RsLOV domain, Richter and colleagues developed a light-controllable version of the endonuclease Cas9, whose function is inhibited under dark conditions. Whether this inhibition is a direct steric effect of RsLOV dimerization on Cas9, or caused by restricted binding of other cellular factors remained open. Illumination with blue light restores Cas9 function, which led to a DNA cleavage in *E. coli* (Richter *et al*. 2013), an approach with the potential to increase the spatiotemporal control of genome editing. Furthermore, optogenetic interaction switches were established that restore the function of split-proteins upon light-induced assembly of the interaction partners. A light-induced dimerization tool of split recombinases based on VVD or Magnets was used to induce DNA modification events, such as the excision of a transcription terminator region and resulting induction of gene expression with blue light within two hours (Sheets, Wong and Dunlop 2020).

Several recent studies highlighted the use of optogenetics to control the intracellular localization of target proteins. An innovative approach applied light-induced phase separation (photoactivated switch in *E. coli*, PHASE) to increase the proximity of enzyme and substrate within the same phase and therefore facilitate enzymatic reactions. The system, based on the blue light dimerization switch CRY2-CIB1 (Liu *et al*. 2008), resulted in up to 15-fold enrichment of POIs and roughly two-fold enhanced rates of enzymatic reactions upon illumination (Huang *et al*. 2020). Even higher on/off rates can be achieved for systems targeting proteins that, at least temporarily, localize to the cytosol, and can therefore be inactivated by sequestration, e.g. to the bacterial membrane. By using the complementary blue light optogenetic interaction switches LOVTRAP (Wang *et al*. 2016) or iLID (Guntas *et al*. 2015) to tether or release an essential cytosolic system component to and from the bacterial inner membrane, almost complete activation or suppression of the bacterial type III secretion system (T3SS) function upon illumination (light/dark ratio of secretion of > 50 and < 0.02, respectively) was achieved (Lindner *et al*. 2020)). This light-induced translocation of effectors by sequestration of endogenous components of the T3SS (LITESEC-T3SS) was used for the light-controlled delivery of pro-apoptotic protein cargos into cancer cells (Lindner *et al*. 2020). iLID-based membrane tethering was also used in an optogenetic protein purification method (mem-iLID) to isolate the membrane-tethered POI with the membrane fraction in the light and then release it by incubation in the dark (Tang *et al*. 2021). Optogenetics is not restricted to the control of intracellular processes, but can also be used to target inter-bacterial or bacteria-cargo interaction. Expression of at least one component of optogenetic interaction switches on the outer surface of the bacterial membrane allowed controlling diverse biological activities. The Magnet system was used by Chen and Wegner to regulate biofilm formation through light-dependent cell-cell aggregation (Chen and Wegner 2017, 2020). In combination with a mercury-activated luciferase (Sciuto *et al*. 2019), this was applied for the sensing of mercury ions (Chen *et al*. 2020). The red/far-red interaction switch PhyB-PIF6 was used for the subsequent binding (upon red-light illumination), transport (by motile bacteria) and release (upon far-red illumination) of extracellular cargo linked to the other part of the dimerization switch (Sentürk *et al*. 2020). The above examples and further optogenetic applications in bacteria are listed in Table 1.

## TOOLS FOR OPTOGENETIC INVESTIGATIONS IN BACTERIA

Optogenetics greatly benefits from specific tools, both for the design and the practical implementation of the experiments. Although most optogenetic setups are tailored to the specific research demands and many labs develop their systems in a do-it-yourself manner (Pouzet *et al*. 2020), bioinformatic resources and physical devices designed to facilitate the investigation of biological processes with optogenetics in smaller environments like bacteria are increasingly being developed. Most resources and databases are open access and easy to adapt to individual demands. One of the most extensive and very intuitive optogenetic databases is optobase.org, which was founded by Weber and colleagues and provides manually curated information about optogenetic systems and their characteristics, publications (searchable by color range, base system or host organism), as well as additional tools such as an application search (Kolar *et al*. 2018). Other recommendable resources are the comprehensive Addgene guide on optogenetics (addgene.org/guides/optogenetics), and the Optogenetic Resource Center by the Deisseroth lab (web.stanford.edu/group/dlab/optogenetics). With the increasing interest in the application of optogenetics in bacteria, the toolbox of technical resources that are specific to or particularly useful in supporting such investigations has also been expanded. One of the first such platforms was presented by Gerhardt and colleagues, who engineered a light plate apparatus (LPA) to illuminate different culture setups in a 24-well plate format (Gerhardt *et al*. 2016). This setup was later improved by integrating microfluidics and an automated closed-loop feedback control system that allows independent control of samples in a 96-well plate (Soffer *et al*. 2021). Optogenetic control of bacteria down to the single-cell level was achieved by combining microfluidics with fluorescence microscopy in a computer-interfaced control setup that can visualize and control dynamic processes for up to 200–400 cells in four to eight fields of view (Chait *et al*. 2017). For larger culture volumes, Steel and colleagues developed Chi.Bio, an open-source platform that provides computational control of light and media parameters as well as temperature settings for the implementation of pre-designed and even feedback-controlled optogenetic experiments with bacteria up to a 25 ml culture scale (Steel *et al*. 2020). Other researchers presented mathematical and computational models that correlate optical inputs with output models like gene expression, allowing to accurately predict the output even for multiplexed systems and in turn to estimate the optimal illumination parameters for a desired output (Olson *et al*. 2014; Olson, Tzouanas and Tabor 2017). Pulsed illumination setups (Hennemann *et al*. 2018) and frequency modulating oscillation (Mahajan and Rai 2018) further support the fine-tuning of optogenetic systems. To bring light into living tissues, a crucial prerequisite for many biomedical applications of optogenetics, photoactivatable printed hydrogels or nanocarriers were developed, which can also be incorporated into the human body and guide light signals to certain sites of action. This technique allowed the remotely light-controlled drug expression and secretion by *E. coli* encapsulated in a hydrogel (Feng *et al*. 2020) and has been applied to treat ulcerative colitis (Cui *et al*. 2021) and for repression of subcutaneous tumors (Yang *et al.*^2020^).

## CONSIDERATIONS FOR THE SUCCESSFUL APPLICATION OF OPTOGENETICS IN BACTERIAL SYSTEMS

The vast majority of optogenetic applications have been developed in eukaryotic systems. In the following, we therefore want to present core considerations for the successful implementation of optogenetics in bacteria. We will discuss specific limitations and challenges of optogenetic applications in bacteria and highlight areas of potential future applications of optogenetics in microbiology beyond currently established research tools. With this section, we want to inspire and support microbiologists to develop individual optogenetic setups and experiments for their specific biological question.

### Light as a trigger for biological processes

Using light to control biological processes in bacteria comes with new challenges and considerations. To apply optogenetics to control biological processes with a high spatiotemporal resolution and dynamic range, specific aspects have to be taken into account, including the kinetics, directionality and sensitivity to ambient light of different optogenetic systems, as well as the wavelength-dependent penetration depth and possible phototoxicity. The choice of a suitable base system is therefore an important consideration.

#### Kinetics and directionality of optogenetic systems

Most optogenetic systems return to their ‘dark’ state within seconds to minutes after illumination ceases (Fig. 1). On the one hand, rapid deactivation of the system in the dark can be beneficial, for example in clinical studies where the bacteria need to be activated at a specific site, but relocation of the activated bacteria would lead to side effects. On the other hand, this feature can complicate long-term studies. Accordingly, the required duration and frequency of illumination is a key variable of different optogenetic base systems and an important factor for their successful application in bacteria. Constant illumination protocols are easier to set up and are mostly used to provide the highest possible binding/unbinding range and therefore achieve a strong on/off ratio. Pulsed illumination setups have the advantage of varying the optogenetic output by modulating light pulse-width or amplitude (Hennemann *et al*. 2018; Dietler, Stabel and Möglich 2019). In combination with a feedback control software, which measures system output like expressed protein levels, variation of illumination can be used to balance bacterial growth and bioproduction in order to maximize long-term yields (Endo and Ozawa 2017; Pouzet *et al*. 2020). Pulsed illumination can also decrease the risk of phototoxic effects on bacterial cultures. However, when applying pulsed illumination setups, users have to consider the fast off kinetics of some optogenetic systems (Fig. 1). Pulsed illumination with longer intervals can sometimes relax the control over biological processes, compared to constant illumination. Therefore, it is recommendable to screen for the best-suited illumination for each setup. The direction of the optogenetic switch also influences the need for illumination. In many settings, triggering an event by (short) illumination will be preferable to triggering it by darkness. Depending on the desired output, optogenetic switches that either dissociate or associate upon illumination are therefore preferable in such cases (see chapter “Addressing specific research questions with direct optogenetic control" for details).

#### Sensitivity to ambient light

Ambient light during cultivation and handling may partially activate optogenetic systems (Ochoa-Fernandez *et al*. 2020). Even the relatively low level of laboratory light can lead to residual activation of sensitive systems. In the few studies that explicitly tested the influence of ambient light, both iLID (Lindner *et al*. 2020) and VVD (Sheets, Wong and Dunlop 2020) were only activated to a low degree by ambient light, while LOVTRAP displayed stronger background activation (Lindner *et al*. 2020).

#### Phototoxicity

High-energy light at shorter wavelengths ranging from UV to blue can have damaging effects on eukaryotic cells (Gentile, Latonen and Laiho 2003) and many bacterial species (Yin *et al*. 2013; Pereira *et al*. 2014). Studies have shown an influence of blue light on growth for model organisms like *B. subtilis* or *P. aeruginosa* (De Lucca *et al*. 2012; El Najjar *et al*. 2020), as well as the light-induced production of carotenoid pigments in *Myxococcus xanthus*, induced by blue-light-generated singlet oxygen (Burchard and Dworkin 1966; Burchard and Hendricks 1969; Galbis-Martínez *et al*. 2012; reviewed by Padmanabhan *et al*. 2021). In most studies, however, the relatively low light intensities required for the activation of optogenetic systems did not influence bacterial growth, division and specific function. Nevertheless, this should be tested for each individual case.

The varying penetration depth of light of different wavelengths is worth considering as well, especially for the application of optogenetics in bulk volumes or in clinical samples. Red and near-infrared light generally penetrates biological tissues well (Cheong, Prahl and Welch 1990; Stolik *et al*. 2000; Jacques 2013) and is less absorbed by common growth media, which is advantageous in these cases.

### Direct control of biological processes by optogenetics

While the application of optogenetics to control bacterial gene expression is becoming an established method, future applications can aim at a more direct protein-based control of biological processes (Brechun, Arndt and Woolley 2017) with widespread application potential: most proteins can in theory be controlled by structural changes, dimerization events (e.g. split-protein domains) or control of localization through optogenetics.

The most widely used tool to control the function of initially non-light-dependent proteins or complexes are optogenetic interaction switches, which can be used to control the binding of a POI, fused to one domain of the interaction switch (the bait), to the other domain (the anchor) upon illumination. Some proteins are activated at a specific cellular localization, for example at the membrane (Levskaya *et al*. 2009 as an example in eukaryotes), or at the bacterial poles (Laloux and Jacobs-Wagner 2014). More often, forced relocation (sequestration) will inactivate the target protein or a complex that requires its presence. Release of the POI can then re-activate the target function. Prerequisites for this approach are (a) the functionality and stability of a fusion of the POI to one of the optogenetic interaction domains, (b) an at least temporary presence of the POI in the cytosol to allow for sequestration and (c) a loss of function of the POI when tethered to the anchor protein. The latter is usually the case for proteins with an essential reaction/interaction interface that is blocked by the interaction with the anchor, or a specific site of action (e.g. as part of a protein complex with a specific localization) (Lindner *et al*. 2020). In eukaryotic systems, anchor proteins can be recruited to the cell membrane (Gil *et al*. 2020) or to organelles like mitochondria (Wang *et al*. 2016), the endoplasmic reticulum (He *et al*. 2017) or peroxisomes (Spiltoir *et al*. 2016). Bacteria are much less compartmentalized than eukaryotic cells; the primary target for optogenetic recruitment of cytosolic target proteins is therefore the bacterial membrane (Lindner *et al*. 2020; Tang *et al*. 2021).

### Addressing specific research questions with direct optogenetic control

Compared to the optogenetic control of transcription, direct (post-transcriptional) optogenetic control requires a few additional considerations. In this paragraph, we want to delineate key aspects for planning and implementing experiments to directly control localization and activity of a POI by optogenetics. While some of these points also apply to controlling transcription, readers who are specifically interested in this aspect should also refer to the information listed in Fig. 1 and the recent review by Baumschlager and Khammash (Baumschlager and Khammash 2021).

#### Does the POI tolerate fusions, and where?

The size of optogenetic interaction domains differs considerably (see Fig. 1 for details). For hetero-dimer interaction switches, the choice of which interaction domain is fused to either the POI or a localization target (e.g. a membrane anchor) may influence the success of the experiment. In most existing studies, the smaller interaction domain (e.g. Zdk1, SspB_Nano, PIF) was fused to the POI (which may be sensitive to sterical hindrance by larger fused domains) and acts as a bait; accordingly, the larger interaction domain (AsLOV2, iLID, PhyB) was used as the anchor domain (Toettcher *et al*. 2011; Wang *et al*. 2016; Lindner *et al*. 2020; Tang *et al*. 2021). In line with this reasoning, Tang and colleagues did not observe binding of the optogenetic interaction switch, when the smaller interaction domain SspB was used as a membrane anchor and the larger interaction partner iLID was expressed as the cytosolic part (Tang *et al*. 2021). While many interaction domains can be freely combined at both termini, the orientation of the protein fusion may also influence the success of optogenetic control. As an example, Toettcher and colleagues noted that for the PIF6/PhyB switch, the larger interaction domain PhyB works best as an N-terminal fusion component, whereas the smaller domain PIF6 does not show any preference for N- or C-terminal fusions (Toettcher *et al*. 2011; Tang *et al*. 2021). Short, flexible peptide linkers (often 5–20 amino acids long and glycine-rich) between the interaction domain and the POI are frequently used to increase the chances to retain function of both the interaction switch and the POI. In any case, the expression level and stability of the fusion protein should be tested by Western blot, as flexible linker domains are subject to proteolysis in some bacteria.

#### Where should the POI be anchored, and should this happen in the light or the dark?

The incorporation of the anchor domain requires the fusion to a protein or motif that confers the desired location. For membrane tethering, an easy and robust option is to attach the anchor domain to an N-terminal transmembrane helix (Lindner *et al*. 2020; Tang *et al*. 2021). As for the bait protein, linker domains can increase the binding capacity of the anchor, and expression and stability should be confirmed. Obviously, the direction of the switch is important. Most optogenetic interaction switches have a dark ground state that can be influenced by illumination, and then recovers over time in the dark. Fortunately for users, systems that associate and that dissociate upon illumination both exist (e.g. iLID or Magnets for association and LOVTRAP for dissociation, see Fig. 1), which even allows to create parallel systems for light-dependent activation and deactivation of a target.

#### How fast does the switch need to occur, how stable should the switched state be, and is light-regulated switching in both directions required?

The kinetics of optogenetic control depend on the base system and the dynamics of the target system. Most optogenetic base systems react to light within a very short time (seconds and below), while their recovery in the dark differs widely, ranging from seconds to hours (Fig. 1). In several cases, mutant libraries with varying binding affinities have been developed, e.g. for the LOVTRAP and iLID systems (Wang *et al*. 2016; Zimmerman *et al*. 2016; Tang *et al*. 2021), which greatly improves both the range of possible applications and the possibility to troubleshoot and optimize applications (see below). If active bidirectional photoswitching is required, phytochrome- or cyanobacteriochrome-based systems (such as PhyB/PIF or Cph1), where association and dissociation of the interacting proteins are induced by illumination with different wavelengths, need to be used.

#### Dynamic range

Optogenetic interaction switches display affinity changes between the light and dark state; no switch features complete vs. non-existing binding in the respective states. The sequestration of a target protein to the membrane anchor therefore depends on expression levels and binding affinities of the interaction partners. While a low anchor/POI ratio or low affinity of the interaction domains may prevent the sequestration of a sufficient fraction of the cytosolic POI in the binding state, too much anchor or high affinity to the POI may lead to insufficient cytosolic concentrations of POI even in the unbinding state, and subsequently affect bacterial fitness or function of the POI. Determining the ‘sweet spot’ of anchor/cytosolic bait expression ratio is therefore instrumental for tight control over downstream processes within optogenetic dimerization switch applications (Lindner *et al*. 2020). Notably, there is an upper limit to the number of bait proteins that can be sequestered. In one of our studies, a protein present in more than 1000 copies could be efficiently membrane-anchored at an anchor expression level that could still be increased > 4-fold without visible detrimental effect on the bacteria ((Lindner *et al*. 2020) and unpublished), suggesting an upper limit of at least several thousand bait proteins. However, this number will strongly depend on the optogenetic base system used, the expression system and of course the host bacterium. For proteins with a large copy number, phase separation-based systems (Huang *et al*. 2020) might offer an alternative, although their capacity and dynamic range remain to be determined. While suitable pre-experiments (see below) can help to estimate the dynamic range of a base system and anchor in a given bacterial species, adjustments to the final experiments may be required for best results (see ‘Troubleshooting and optimization’ below). Notably, such adjustments are greatly facilitated by a tunable anchor expression system and a base system featuring versions of different affinity. For example, efficient sequestration of a natively expressed cytosolic protein to the membrane was only possible using the V416L version of the LOVTRAP *As*LOV2 protein (Kawano *et al*. 2013; Lindner *et al*. 2020), while screening and even directed mutagenesis of iLID SspB versions allowed Tang and colleagues to optimize light-controlled protein purification (Tang *et al*. 2021)

#### Are there any restrictions to the wavelengths that can be used?

While most bacteria appear to tolerate the low light intensities required for optogenetic experiments well, sensitive species can be harmed, especially by blue light. When working with these species or for experiments in animal tissue or bulk volumes, red- to infrared-sensitive systems may be preferable due to the lower photon energy and (generally) deeper penetration of light in this part of the spectrum (also see “Light as a trigger for biological processes", above). For experiments involving additional fluorescent proteins or dyes, the compatibility of their excitation/emission wavelength with the optogenetic control also has to be taken into account. For example, optogenetic systems activated by blue light should not be combined with GFP: the excitation wavelength of GFP (∼488 nm) overlaps with the activation spectrum of most blue-light-activated systems (often a broad peak from 400–500 nm, e.g. for LOV2 (Salomon *et al*. 2000)), which could lead to cross-activation of the optogenetic system upon analysis of the fluorescent protein. Similarly, red fluorescent proteins such as mCherry (excitation and emission peak at ∼587 nm and ∼610 nm, respectively) may overlap with cyanobacteriochrome- and phytochrome-based optogenetic systems. Studies have therefore mostly used mCherry as a read-out for green light-controlled optogenetic systems (such as Magnets (Baumschlager, Aoki and Khammash 2017; Chen and Wegner 2017), iLID and LOVTRAP (Lindner *et al*. 2020), and Cry2-CIB1 (Huang *et al*. 2020)) and GFP for red-light-controlled systems (e.g. PhyB-PIF (Sentürk *et al*. 2020)).

#### Requirement and availability of cofactors

Optogenetic switches that are based on cryptochromes, BLUF- or LOV-domains require flavin cofactors (FMN, FAD) that are ubiquitous and present in sufficient concentrations in most bacteria (Entsch and Ballou 2013). Other cofactors like phycocyanobilin (PCB) or biliverdin, which are needed for optogenetic switches based on phytochromes or cyanobacteriochromes are not necessarily present in bacteria and often have to be co-synthesized (Ma *et al*. 2020; Uda *et al*. 2020) (Fig. 1). In the case of phycocyanobilin (PCB), a cofactor for several systems in the red/far-red range, this is ensured by the integration of two synthesis enzymes Ho1 and PcyA, which catalyze the transformation of protoheme to biliverdin and its subsequent reduction to phycocyanobilin, respectively (Gambetta and Lagarias 2001; Ma *et al*. 2020). It was shown that PCB can affect the biomass of some bacteria at higher concentrations (Raghavan, Salim and Yadav 2020), suggesting toxicity, which should be considered for the respective systems.

#### Preparatory experiments

To establish an optogenetic system and estimate its parameters in a given host bacterium, it is recommendable to perform preliminary experiments. As an example, the binding of a (compatible) fluorescent bait protein to the membrane anchor can be used to test the function of the system for a given anchor construct, expressed at different levels. The dynamics of tethering and release events can then be directly visualized via fluorescence microscopy (Lindner *et al*. 2020). Preparatory experiments are especially useful prior to complex, costly, and/or long-term experiments, and may be skipped otherwise.

#### Troubleshooting and optimization

The main variables for troubleshooting and optimization, both for the preparatory and main experiment, are (i) illumination settings, (ii) the anchor/bait ratio, and, if possible, (iii) variants of the base system with different affinities for the anchor. While problems arising from incomplete binding of the interaction partners call for stronger illumination, higher anchor/bait ratio, and/or the application of variants with higher affinity, the opposite problem, detrimental binding under ‘off’ conditions, may be counteracted by lower (or pulsed) illumination, lower anchor/bait ratios, and/or less affine protein variants.

**Figure 3.**
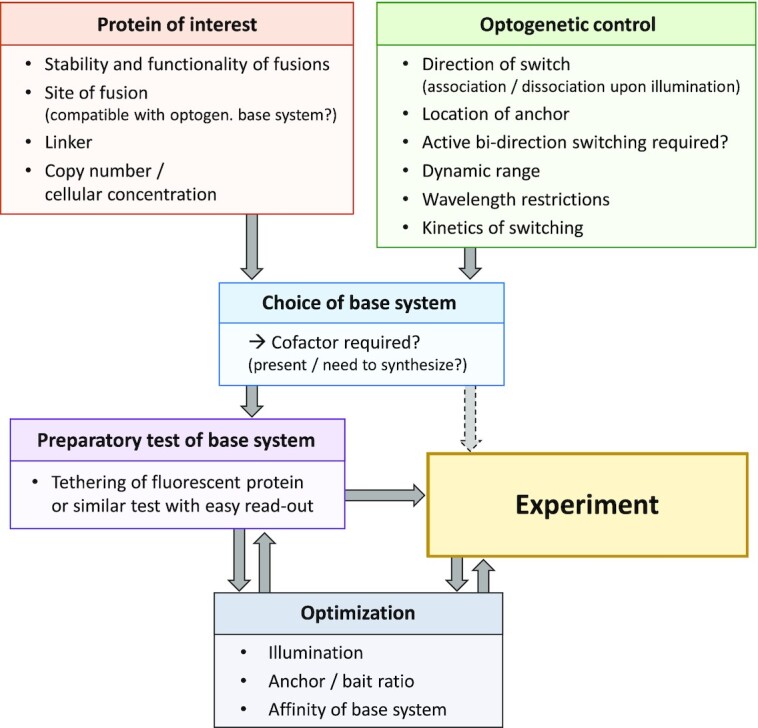
Considerations for experiments using direct optogenetic control See main text for details.

Fig. 3 summarizes a suggested workflow and main considerations for direct optogenetic control experiments.

## BEYOND THE HORIZON—FUTURE OPPORTUNITIES OF OPTOGENETIC APPLICATIONS IN BACTERIA

After a slow start, the application of optogenetic principles to control bacterial functions is gaining speed rapidly. A broad PubMed search for ‘optogenetics bacteria’ already brings up 407 hits, more than half of which have been published in the last four years. Similarly, a search for ‘host:bacteria’ in the manually curated OptoBase database yields 92 papers, 66 of which have been published since 2017.

While optogenetic control of gene expression and, increasingly, the direct control of protein function by light have been established in bacteria, other approaches that were put forward or already implemented in eukaryotes have not yet been applied in prokaryotes. In this last paragraph, we want to highlight some examples to demonstrate the widespread potential of optogenetic applications in bacteria and potentially inspire readers to think beyond the horizon.

Eukaryotic optogenetic applications that aim at light-dependent protein degradation by proteasome recruitment (Renicke *et al*. 2013; Baaske *et al*. 2018) have been established for years and could be considered for optogenetic applications in bacterial species like *Mycobacterium* or *Streptomyces*, which also carry proteasomes (Becker and Darwin 2017). Furthermore, the development of light-controlled proteases based on the bacterial SsrA-SmpB system (Karzai, Roche and Sauer 2000) or the direct influence on degron systems, where a protein degradation sequence (degron) is caged by a photoactivatable domain (Usherenko *et al*. 2014; Mondal *et al*. 2019), could be considered. For the direct optogenetic control by sequestration of cytosolic proteins, the bacterial membrane was so far used as the main sequestration point, due to its large surface and relative ease of anchoring of proteins (Lindner *et al*. 2020; Tang *et al*. 2021). Applying phase separation (Huang *et al*. 2020) may provide an alternative, although so far, the dynamic range of activation/deactivation was relatively low. Future applications can also consider the light-dependent recruitment of a target protein to subcellular compartments such as polar protein structures like PopZ or HubP (Surovtsev and Jacobs-Wagner 2018). To overcome the limited range of blue light in tissues or large cultures, upconversion nanoparticles that can convert near-infrared to blue light (Chen *et al*. 2014) might be used for the application of blue light-controlled systems (which make up the majority of both optogenetic base systems and applications) by far-red light. There are still only few examples for engineered intramolecular optogenetic systems beyond the TCS used for transcriptional control (Lee *et al*. 2008; Wong, Mosabbir and Truong 2015; Jones, Mistry and Tavassoli 2016). Future applications could aim at controlling signaling cascades by modifying different kinases (Leopold, Chernov and Verkhusha 2018) or phosphatases (Hongdusit *et al*. 2020; Hongdusit and Fox 2021). Arroyo-Olarte and colleagues proposed to investigate host-pathogen interactions using optogenetic control, such as a modified inositol phosphatase which might alter the host phosphoinositide level by light during infection processes like induced phagocytosis caused by *Yersinia* or *Listeria* (Arroyo-Olarte *et al*. 2018). A review collection of optogenetic applications in eukaryotes that cover subcellular organization (Kichuk, Carrasco-López and Avalos 2021), signal transduction (Mühlhäuser *et al*. 2017) or widespread utilizations and design strategies (Brechun, Arndt and Woolley 2017; Endo and Ozawa 2017; Oh *et al*. 2021) can also be considered as an information and inspiration base for new developments in bacteria.

Given the great potential of optogenetics, its obvious advantages for non-invasive, highly precise interventions, and the ever-expanding toolkit, it will be exciting to watch the development and use of optogenetic systems in prokaryotes to improve both our understanding of bacterial processes and their application in basic research, biotechnology, and medicine.

## Footnotes

1 The photocaged tyrosine was introduced by amber stop codon suppression, which requires the presence of additional enzymes and external addition of *ortho*-nitrobenzyl tyrosine
